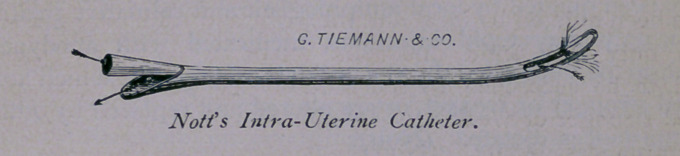# On a Few Instruments for the Practical Treatment of Uterine Disease

**Published:** 1871-07

**Authors:** Philip Adolphus

**Affiliations:** 169 Dearborn Street; Chicago


ARTICLE III.—*On a few Instruments for the Practical Treatment of Uterine Disease.* By PHILIP ADOLPHUS, M.D., Chicago.

(Fourth paper, continued from the May Number.)

1st. Emmet's Applicator, used to apply medicated solutions to the cervix and uterine cavity.

2d. Nott's Intra-Uterine Catheter.

Every one who has handled the perfect instrument, known as Emmet's applicator, is aware of its value. It fulfills the several uses of cleansing the cavity of the cervix and body of the uterus of secretions, and of applying medicated solutions to these parts, with admirable certainty.

Its fitness for the former purpose has been discussed in a previous paper; in this communication the writer will impress the reader with its value, when the medication of this organ is proposed.

Every instrument that is to be used in the uterine cavity, must possess a calibre permitting an easy entrance, and ductility, combined with the requisite firmness. We have seen that Emmet's probe has these qualifications, and that his applicator is a *fac-simile* of this instrument,—a little larger, slightly stouter, and flat at its extremity, without the bulb of the other instrument. Made of annealed silver wire, it is capable of assuming every conceivable shape which the hands of an experienced operator may impart to it, and is mounted on a light wooden handle.

A slide, consisting of a compressed coil of brass wire, five inches in length, hollow and flexible, resembling an enlarged watch spring, capped at each extremity by a hollowed bead of gutta-percha, passes over the probe, and acts when the latter is armed with medicated cotton wool, and the coil is propelled towards its point by pushing the roll of cotton off into the neck or cavity of the uterus.

*Emmet's Applicator.*

It has the following advantages over all instruments used for similar purposes, with which the writer is acquainted: of permitting the introduction of medicated solutions into any part of the cavity, and of depositing them instantly when the parts are reached.

It is convenient to possess probes (applicators) of different lengths, suitable to enter the cavity of the cervix or fundus; one slide will be sufficient for all.

The manner of using the applicator is as follows: Slip the coil over the probe, and arm it with cotton wool. Place patient in the usual position, (semi-prone, when using Sim's Speculum; on the back, when Nott's Speculum is applied) the speculum being in situ, steady the cervix with Nott's Vulsellum, introduce Emmet's probe for the purpose of ascertaining the length, direction and sensitiveness of the uterine cavity; remove it; give the applicator the identical curve which was imparted to the probe, and introduce it, (armed with cotton and moistened with warm water), in the same direction and with equal care, into the uterine cervix or cavity. Remove the applicator now charged with the secretion of the cavity, push the coil or slide towards the end of it, the fouled cotton still adhering will then drop off. This procedure cleanses the parts of accumulated secretions, and prepares them for medication.

Again introduce Emmet's probe as a guide (a very important precept, if you wish to prevent bungling and consequent pain), follow it, on its removal, by another applicator already armed and moistened with the necessary medicated solution, (care must be taken not to *saturate* the cotton wool, else the solution will run into the vagina—a result to be deprecated), insert this into the womb, and whilst sliding the coil towards the cervix with the left hand, withdraw the probe in an opposite direction with the right hand; this manœuvre results in the introduction of a piece of cotton wool, from one to four inches in length, into the uterine cavity, where it may be left in safety, provided the solution is weak.

Much mischief may be done with this instrument as with other caustic holders; for concentrated solutions, as well as liquid caustics, may be applied by it, with ease. The applicator merely places a roll of cotton in the cavity of the uterus; whether the *agent* with which it is charged shall induce benefit or mischief, will depend upon the operator.

This is especially the case with those solutions which do not coagulate or form inert albuminates with the secretions of the cavity. Articles which form these coagulates, when introduced into the cavity smeared with tenacious mucous discharges, are not so dangerous; for these protect the walls from their caustic effect, and preserve the organ in despite of the operator. Far otherwise is it when the heavy muco-purulent covering has been removed, and a denuded, mucous surface, with exposed bloodvessels and capillaries (Nott) receives the caustic application: collapse, general peritonitis, etc., may take place, and death, by metro-peritonitis, has been the result of an application of a solution of ʒj of nitrate of silver to ℥j of water, by means of a camel's hair pencil, to the interior of the womb, as related by Noeggerath.*

In order to determine the advantage of intra-uterine applications, we should study the symptoms of chronic uterine affections, uninfluenced by remedial measures.

Thus we find in these cases, abnormal congestion resulting generally from cold taken during menstruation, and leading to acute uterine catarrh (Klob), which forms the sole and principal condition of many cases of dysmenorrhœa (Tilt).

We have, furthermore, affections dating back from former pregnancies and abortions, which profoundly affect the economy. Exciting causes, such as fatigue, excitement, alarm, and change of temperature, often superinduce increased irritation and hyperæmia, which, happening at or about the monthly periods, result in cellulitis, peri-metritis, ovaritis, salpingitis and general peritonitis.

Will the application of fluid and liquid caustics, under these circumstances, be beneficial? Will they not rather produce these complications? Again, if, after these applications, the patient complains of the painful symptoms so graphically described by authors, as sometimes accompanying medication by caustics, and recovers therefrom; has this result been effected by these caustics, or has not rather the inherent force of her constitution saved her from the effects of injudicious treatment?

The writer concludes, from the results of his observations, that *mild applications, judiciously used, are not merely safe but curative.*

* E. Noeggarath's Contributions to Midwifery and Uterine Pathology. 1858, fol. 30.

Furthermore; that caustics to the interior of the womb are hazardous remedies in able hands, but inadmissible in those of the inexperienced and rash.

Much interest has of late been manifested in the discussion of the question of intra-uterine medication, and it has been examined in all its bearings by Kammerer, Nott, Peaslee and Lente. (See papers published in the New York Medical Journal, and the American Journal of Obstetrics). Many other distinguished men have also expressed their views, either for or against the application of concentrated solutions and caustics to the uterine cavity; but while their views are at variance, there is no doubt that the tendency of the practice of many of the leaders of the profession is towards the abandonment of solid caustics and concentrated caustic solutions, as applications for the interior of the womb.*

In connection with the use of the applicator, it will be proper to state that applications of various strengths have been and are constantly applied for erosions, excoriations and ulcerations of the cervix uteri, by the profession at large. This is considered by the writer erroneous practice. For these simple excoriations and erosions are certainly not more painful than the same lesions on the lip or buccal cavity; they exist in almost every healthy woman, and are neither causes of complaint or treatment, and do not come under our cognizance as such. "When an erosion exists on the cervix, I believe that it is almost without an exception but a cropping out of the diseased condition above, or an excoriation from the uterine discharge constantly bathing the parts." † "Erosions are generally accompanied with acute and chronic catarrh

* Surgery of the Cervix. T. A. Emmet. American Journal of Obstetrics, Feb., 1869, fol. 342.

The Treatment of Endometritis by Uterine Injections. I. C. Nott. American Journal of Obstetrics, Nov., 1869, fol. 470.

I. C. Nott. Intra-Uterine Medication. N. Y. Med. Journal, June, 1870; and American Journal of Obstetrics, May, 1870.

Intra-Uterine Medication. E. R. Peaslee. N. Y. Medical Journal, July, 1870.

Prolapsus Uteri; its chief Causes and Treatment. Thos. Addis Emmet. N. Y. Med. Record, May, 1871, fol. 98.

† T. A. Emmet. Op. cit., fol. 97.

of the uterus and vagina, and are caused either by this or other forms of intumescence of mucous membrane." (Klob, p. 239).

"Granulating ulcers rarely exist independently, but are generally combined with chronic uterine affections."* Clinical experience convinces him that the mucous membranes of the uterus in all its parts, are frequently simultaneously and consecutively affected, and that lesions of the cervix co-exist and are superadded. These views are held by Scanzoni, West, Hewit, Klob and other men of eminence. Moreover, the division, by authors, of these diseases, into affections of the mucous membranes of the body and cervix, and affections of the parenchyma of the body and cervix, cannot, in old standing cases, be distinguished clinically.

Therefore, a general unbiassed view of each case, based on its history and on pathological anatomy, should be always attempted, and the treatment based thereon; for it is not logical to apply local treatment to the cervix exclusively, when the body and fundus are equally implicated.

Another point of great practical importance in the treatment of the sexual organs by local applications, which has not, I believe, even been alluded to by writers, is, that different modifications of treatment in like diseases, must be adapted to the varying circumstances of each patient. Hospital patients, the wealthy, and those having leisure, can bear, *ceteris paribus*, more powerful applications for the same diseases; whilst like applications would be inadmissible in patients who visit the office, or those whose circumstances prevent their resting after treatment.

This appears self-evident, and is consequently taken for granted, yet in practice it is constantly neglected.

Nott's Intra-Uterine Catheter, the instrument now to be described, is used for the double purpose of cleaning the cavity of the uterus of secretions, and of medicating it.

It is simply a double current catheter, made of silver, nine inches in length, and constructed in such a manner that the introduced fluid, injected through a narrow tube, not more than one-fifth the calibre of the catheter (situated along its concave wall), passes into the cavity of the womb; rushes out, not merely

* Pathological Anatomy of the Female Sexual Organs. Julius M. Klob, 1868.

alongside the catheter, where it partly escapes from the os uteri; but it also escapes through a wide canal in the convex portion of the instrument, whence it emerges at the opening. The egress of fluids is facilitated by an aperture, an inch in length, on each side of the catheter (the eyes of the instrument).

*Nott's Intra-Uterine Catheter.*

The instrument is used in the following manner: The patient having been placed on her back or side, the proper speculum is introduced (Nott's or Sim's), Emmet's probe ascertains the direction of the womb, Nott's catheter is now gently applied to the os, and introduced. If it does not enter easily, no force being permissible, the cavity of the cervix is dilated by Nott's dilator, in the manner described in a former paper. After its introduction, a syringe filled with warm water is inserted into the orifice of the catheter, and its contents gently expelled. This is repeated, until the return current emerging from the womb looks clear, showing that the cavity of the uterus is freed of secretions. The syringe is then again filled with an astringent, detergent or sedative solution, and its contents injected. The catheter is removed, a glycerine suppository is left near the os uteri, and the speculum withdrawn.

In the American Journal of Obstetrics, of February, 1869, is a translation by Dr. Joseph Kammerer, of an article by Dr. J. Cohnheim, Berlin, 1868, entitled "Historical Review of Uterine Injections;" which is exhaustive. It contains extracts of the entire literature of intra-uterine injections up to 1868. Having examined everything accessible on that subject, that had been written up to date; and having, since the introduction of the Intra-Uterine Catheter, by I. C. Nott, of New York City, to the profession in November, 1869, injected the uterine cavity over three hundred times, with tepid water and mild astringent fluids, without pain or other disagreeable manifestations to the patient at the time, or afterwards; the writer merely repeats what has been so well said by Thomas, Tilt, Peaslee, Kammerer, Noeggerath, but especially by Nott, that *intra-uterine injections are*

*dangerous whenever in their performance the following prerequisites are disregarded:*

1st. *There must be a non-irritable uterus.* Whenever bipalpation or gentle probing produces pain, even gentle ablutions with tepid salt water must be held in abeyance until the parts have been quieted by local antiphlogistic and calmative treatment; then only may tepid injections, medicated with anodynes, be tried, to be succeeded by the mildest detergents, which are rendered stronger or weaker, or are altered and replaced by others, as circumstances dictate. (Nott).

Should we neglect this imperative rule, intense pain (uterine colic) or sudden collapse is the immediate result, and peritonitis, haematocele, ovaritis, etc., follow in a few hours. "The uterus, like the peritoneum, the periosteum and bones, is insensible in its normal state, but exquisitely sensitive when inflamed." (Nott on Intra-Uterine Medication. American Journal of Obstetrics, May, 1870, fol. 58.)

2d. *There must be present an open os, canal of cervix, and internal os;* if these are not patent, they must be opened by sponge and laminaria tents, incision or dilators. Neither of these can be used when irritability of parts is present; this should be allayed by the means mentioned above. (Nott, Peaslee and Kammerer.)

3d. *A proper instrument is indispensable.* Whatever instrument is used, the injected fluid should be permitted to flow away with ease through the instrument itself, or by the side of it. Authors use various instruments: Peaslee, Kammerer, Noeggerath, Scanzoni and Kiwish use syringes; Tilt and Nott, catheters; with the latter instrument were performed all the injections of the writer.

4th. *The vehicle should be of proper temperature.* Water and other fluids of lower temperature than the body have often produced painful contractions of the uterus; therefore it should be injected slowly, of the heat of the body. Moreover, care should be taken not to inject a liquid which will coagulate in the cavity of the uterus, or in the tube of the catheter; the precaution of carefully examining the instrument immediately before injection and after it, in order to insure its patency, is self-evident; immediate removal of the instrument becomes imperative whenever mucus or blood obstructs either of the tubes.

The double current catheter of Nott cannot distend the uterus, (for the fluid discharges itself as rapidly as it enters), therefore the question, how much or how little should be injected for the purpose of cleansing and medication is not relevant.

To these precautions the writer adds:

5th. That caustic solutions are inadmissible, and must not be used; for they are liable to produce immediate and remote symptoms, prejudicial to the patient and discreditable to the physician. Furthermore, on emerging from the uterus, they collect in the vagina and act mischievously. The writer is aware that eminent men use caustic applications, but they also recount symptoms and phenomena occurring during intra-uterine medication, which he has not experienced when using a graduated course of dilute medication.

The use of Nott's intra-uterine catheter will, however, always be circumscribed, for the following reasons:

1st. With it, mild solutions must be used exclusively.

2d. In order to insure success, it would require frequent injections, which are not admissible in private practice, for they consume the time of the practitioner and the means of the patient. For hospital purposes this instrument is excellent, and should be adopted extensively.

The great merit of Nott in its introduction, is not merely that he has shown that intra-uterine injections are always safe when the above-named precautions are strictly followed; but that he has proved by experiments that concentrated solutions introduced into the uterine cavity either by injection or ingestion, are dangerous; that they are often apparently harmless, only because the secretions of the cavity protect the uterus from harm; or, because the caustic unites with these secretions, and forms inert albuminates.

The writer states, unqualifiedly, that injections practiced with the precautions herein detailed are devoid of the slightest danger and as harmless in their results, in competent hands, as the simplest operation in surgery. This opinion is based on experimental inquiry, and is therefore worth more than the theoretical dicta of eminent men, who have had no practical experience in its performance, and are therefore not competent to express an authoritative opinion.

(The injections performed by the writer were instituted for the purpose of ascertaining, not so much the necessity and appropriateness of uterine injections in any given case; or their superiority over other methods of intra-uterine medication; as the safety of the operation when all avoidable non-indications were set aside).

Finally, the same principles govern uterine injection with those of uterine ingestion, the same hazards are encountered in both cases by concentrated solutions. The writer prefers, with Peaslee, the use of ingestion; for, with Emmet's applicator, cotton wool charged with mild solutions may be left in the womb until they are discharged, without pain or other detriment to the patient. The time will arrive, thanks to Nott and others, when the mucous membranes of the uterus will be treated like the same membranes in other portions of the body, and when sound anatomical and pathological views will govern the practice of intra-uterine medication.

169 Dearborn Street.

## Figures and Tables

**Figure f1:**
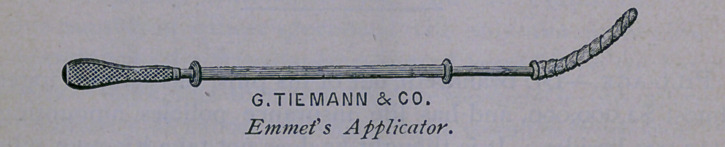


**Figure f2:**